# Comparison of Methods for Cleaning Enteral Feeding Tube Junctions of the New International Standard (ISO 80369-3)

**DOI:** 10.1159/000525367

**Published:** 2022-06-21

**Authors:** Hiroaki Koya, Keiko Ishino, Makoto Kishimoto, Naomi Kurata

**Affiliations:** ^a^Division of Social Pharmacy, Department of Healthcare and Regulatory Sciences, School of Pharmacy, SHOWA University, Tokyo, Japan; ^b^Division of Infection Control Sciences, Department of Clinical Pharmacy, School of Pharmacy, SHOWA University, Tokyo, Japan; ^c^Department of Pharmacy, Kirishima Medical Center, Kagoshima, Japan; ^d^Division of Clinical Nutrition and Metabolism, Department of Clinical Pharmacy, School of Pharmacy, SHOWA University, Tokyo, Japan

**Keywords:** ISO 80369-3, Enteral feeding, Cleaning, Bacteria, EnClean®

## Abstract

**Introduction:**

International standards for enteral feeding involving the use of feeding tubes with junctions have been introduced. If these junctions are not properly cleaned, they can become contaminated, leading to microbial infections. We aimed to compare the ease and effectiveness of cleaning of four methods using the number of bacteria.

**Methods:**

We compared enteral nutrition tube junctions cleaned using four methods such as water, toothbrush, cotton swab, and EnClean® brush with an uncleaned control. Once daily for 7 days, the tubes were injected with nutrients, cleaned, and incubated at 37°C. Samples for bacterial culture were collected before injections on days 7, 14, 21, and 28. The culture samples were incubated at 37°C for 48 h, and the number of colonies was counted.

**Results:**

The number of residual bacteria on day 28 did not differ between the four cleaning methods and the control group. Moreover, no significant difference was observed in bacterial counts among the four wash methods. The number of washes did not differ among cleaning methods.

**Conclusion:**

The bacterial count in the ISO-standardized tube junction increased, and none of the cleaning methods decreased the counts.

## Introduction

In recent years, international standards have been established in the area of enteral nutrition to ensure safety. The introduction of international standards has been studied to improve medical safety. Guidelines on “the introduction of international standards (ISO (IEC) 80669 series) for prevention of interconnection” were issued [1, 2]. Implementation of the ISO 80369-3 (new) standards started in December 2019 in Japan [1, 2].

Enteral administration is a more physiological approach to nutritional support than parenteral nutrition, which is reflected by a lower frequency and severity of metabolic complications [3]. The structure of the new standard product makes it easier for formulas to remain in the connector than that of old standards (online suppl. Fig. 1a, b; for all online suppl. material, see www.karger.com/doi/10.1159/000525367) [2, 4]. Diarrhea is an adverse event associated with enteral nutrition. This may be attributed to the concentration of nutrients and bacterial infections [3]. The main cause of diarrhea in patients receiving nutritional supplements is said to be the temperature and rate of the nutritional supplement, but bacterial diarrhea can also occur depending on the condition of the nutritional supplement [5]. To prevent microbial infections caused by the contamination of the tube junction made as per the new standard, it is necessary to thoroughly clean the junction. Several cleaning methods and devices have been proposed [4]. However, there are no reports comparing the efficiency of these cleaning methods.

Here, we aimed to compare the four clinically usable cleaning methods with no cleaning at all and investigate methods that suppress bacterial growth compared with no cleaning. We experimentally cleaned the junctions of tubes that were infused with nutritional supplements using the four different methods. Bacterial contamination at the tube junction was evaluated and compared for each method. The results of this study may help evaluate the ease and effectiveness of cleaning enteral feeding tube junctions for bacterial control compared with no cleaning.

## Materials and Methods

### Enteral Feeding Model and Nutrient Injection

Table 1 summarizes the instruments and reagents used in the experiments. The model apparatus for the administration of enteral nutrition was constructed as shown in online supplementary Figure 2a. The tube junctions were divided into groups cleaned using four different methods: (1) water, (2) toothbrush, (3) cotton swab, and (4) EnClean® brush (LumaClean, LLC, Doylestown, PA, USA) cleaning. As a control, some tube junctions were left uncleaned.

The experimental design is illustrated in online supplementary Figure 2b. For each cleaning group, three tubes were assessed at each time point: 7, 14, 21, and 28 days (12 tubes in total per cleaning group). Nutrients were passed through all 60 tubes (3 tubes at 4 time points for 4 cleaning groups and 1 control group) per experiment from day 1 to day 7. If all experiments were performed in one period, the number of feeding tubes used in one experimental operation would be high, and the time and physical burden on the experiment are large. With this in mind, the experiment was divided into two periods: experiment 1 from February 13 to March 16, 2020, and experiment 2 from April 15 to May 15, 2020. Three groups were tested in experiment 1 (water, toothbrush, and cotton swab cleaning) and three in experiment 2 (water, EnClean®, and no cleaning). To compare the data from experiments 1 and 2, water cleaning was evaluated during both phases.

All experiments were performed in a safety cabinet. Nutritional supplements were placed in a sterile Petri dish, and 20 mL was weighed using an injector. After weighing, the injector was filled up to the tip of the injector, as shown in online supplementary Figures 1c, and any nutrient adhering to the outside of the injector was wiped off with sterile gauze. After injection, sterile purified water was flushed through the tube using the same injector. Before storing, the joints were cleaned according to each group, and the control group was flushed with water. The tubes were wrapped in plastic, the junction was covered with a lid, and the tubes were incubated at 37°C. Injectors were discarded after flushing the tubes with water. This was repeated once daily for 28 days.

### Cleaning of the New Standard Feeding Tube Joint

In experiment 1, the feeding tube junctions were cleaned using sterile purified water, a toothbrush, or a cotton swab; in experiment 2, cleaning was performed using sterile purified water or an EnClean® brush. Each cleaning round was completed when the practitioner judged that the area was visually clean; the number of rounds of cleaning (referred to as the number of washes) required to achieve this was noted. More specifically, “visually clean” was a visual observation of the following: (1) no nutrient residue is observed, and (2) beige water of the nutrient is not observed in the groove.

#### Water Washing

The tube junction was immersed in water in an unused cup. After manually vibrating for approximately 1 s, it was pulled out of the water; this was counted as one washing. The procedure was repeated, and subsequently, the tube junction was wiped with a sterile gauze.

#### Toothbrush

In this method, cleaning was done with a wet toothbrush. Before use, the toothbrushes were rinsed thoroughly with running water to remove any visible organic matter, disinfected by immersing in 125 ppm NaClO for 1 h and air-dried between 20°C and 30°C. The number of times the tube junction was scrubbed was recorded. The same toothbrush was used for 28 days.

#### Pediatric Cotton Swab (Extra-Fine)

Pediatric cotton swabs were autoclaved prior to use. A dry cotton swab was used for cleaning the tube junction. If the tube junction was not satisfactorily cleaned in one wipe, fresh swabs were used for repeated cleaning; swabs were not reused. A wet cotton swab was used for the last cleaning for rinsing. Any excess water left after cleaning was wiped off with a sterile gauze. The number of swabs used was recorded, and the swab used for rinsing was excluded.

#### EnClean® Brush

The EnClean® brush is a special instrument for cleaning ISO 80369-3-standard tube joints and is sold overseas. Briefly, the EnClean® brush was dipped in water in an unused cup and connected to a tube to be cleaned and twisted once for cleaning. After one wash, the instrument was rinsed with the water in the cup and once again connected to the tube and twisted. This procedure was repeated, and the number of repetitions was recorded. The brush was disinfected and dried as per the manufacturer's instructions and the video provided on the product page (https://youtu.be/dydrX6OPdN8).

#### No Cleaning Group

Tubes that were not cleaned were used as the control group. The nutrients were injected and stored as in the other four wash groups. That is, 20 mL of nutrient was injected, and then 20 mL of sterile purified water was flushed. Subsequently, the tube was closed without cleaning the tube connection, wrapped in plastic wrap, and stored in an incubator at 37°C.

### Collection of Culture Samples

In experiments 1 and 2, the first culture sample was collected from the junction of each tube prior to nutrient injection on day 1 (immediately after opening the packaging). The tube was injected with nutrients, flushed with sterile purified water, and incubated at 37°C for 24 h. This process was repeated for 7 days. The tubes were discarded after sample collection. Similarly, tubes were prepared for repeated nutrient infusion for 14, 21, and 28 days, and each culture sample was collected in the same manner.

Sterile pediatric cotton swabs (extra-fine) were used to collect samples. The tube joint was wiped with a swab, and the swabs were placed in sterile purified water in a cryovial. The swabs were removed, and this water was used as the culture sample.

### Measurement of the Residual Bacterial Count

Bacterial counts were measured using the agar dilution plate method. The number of colonies was counted in the medium (with 100–300 colonies), and the average numbers of colonies calculated from two plates at the same magnification (for example, if a 100-fold diluted plate was found suitable for counting, the average number of colonies was calculated from two 100-fold diluted plates), the dilution ratio was multiplied by 10 (to convert the number per 100 μL to the number per 1 mL), and the number of CFU/mL was recorded. The plates were incubated at 37°C for 48 h, and the number of colonies was recorded. The median based on the average value of the three tubes (belonging to the same group) was used for the analysis.

### Statistical Analysis

The mean number of washes required for each cleaning method was compared using Tukey's test, and the median number of the residual bacteria was compared using the Steel test. The no-washing group in experiment 2 was used as the control. JMP 15.0 (JMP, Cary, NC, USA) was used for the statistical analysis. The significance level for both tests was set at 5%.

## Results

### Ease of Cleaning

#### Water Washing

A large amount of nutrient residue was effectively removed by simply dipping the joint in water and manually vibrating the tube.

#### Toothbrush

It was difficult to clean deep into the groove, even by rotating the brush. Repeated rotations were necessary for effective cleaning. The tip of the toothbrush should be used to clean the joint.

#### Pediatric Cotton Swab (Ultrafine)

The cotton balls of ordinary cotton swabs were larger than the groove widths of the tube joints. Only pediatric cotton swabs (ultrafine) could barely be inserted into the joint. Cleaning the joints was a challenging process as the shaft of the swab would break when it was held far from the insertion point and force was applied. Therefore, it was necessary to hold the swab close to the joint. As the joint of the cleaning tube was threaded, the cotton swab ascended along the screw during one round, and the bottom of the entire joint was difficult to clean.

#### EnClean®

The brush was easy to hold and use, but the bottom of the joint groove was difficult to clean and left an impression of the nutrient residues. The joint had to be washed repeatedly to remove the residues. As per the instruction manual, the cap was closed with oral rinse solution; however, we found that this solution made the brush sticky.

### Residual Bacterial Counts after Incubation

**Fig. 1:**
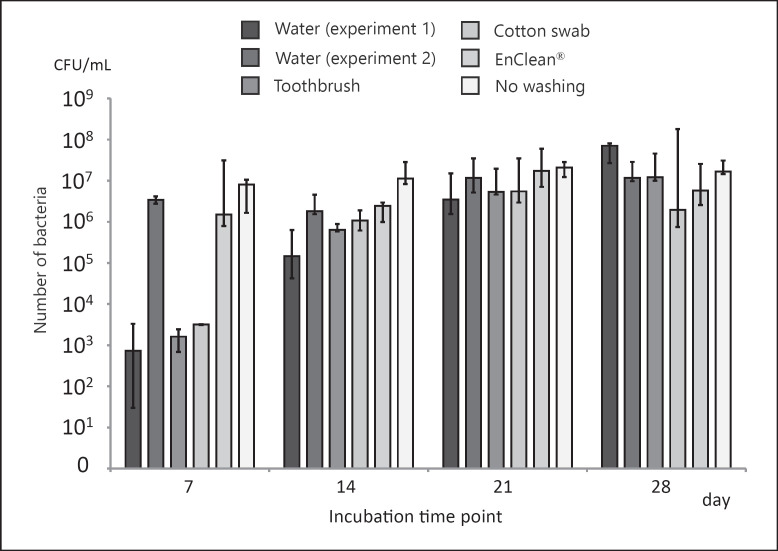
Residual bacterial counts. The number of residual bacteria after each washing method at each culture time in experiments 1 and 2. The *y*-axis represents the logarithm of the number of bacteria. The *x*-axis shows the sampling day. The error bars represent the maximum and minimum values.

In experiment 1, the median residual bacteria count in the tube junction on day 28 was 7.1 × 10^7^ CFU/mL in the water rinse group, 1.2 × 10^7^ CFU/mL in the toothbrush group, and 2.0 × 10^6^ CFU/mL in the pediatric swab group. Moreover, the median residual bacteria count in experiment 2 was 1.2 × 10^7^ CFU/mL in the water rinse group, 5.8 × 10^6^ CFU/mL in the EnClean® group, and 1.7 × 10^7^ CFU/mL in the control group. The bacterial colony counts at the tube junction did not differ significantly between the tubes that underwent cleaning and those in the control group after 28 days (Fig. 1) (water experiment 1: *p* = 0.5, water experiment 2: *p* = 0.8, toothbrush and cotton swab: *p* = 0.9, EnClean®: *p* = 0.8).

### Number of Washes

The median number of washes was calculated for the three tubes in each wash group after 28 days of injection. Washing with water in experiment 1 required the least number of washes (1.7 ± 0.7), whereas the toothbrush washing required the greatest number of washes (4.7 ± 1.1). Moreover, washing with water in experiment 2 required 2.8 ± 0.7 washes. Cotton swab washing required 3.1 ± 1.3 washes, whereas EnClean® washing required 4.1 ± 2.0 washes. No significant difference was observed between water washing in experiment 2 and cotton swab washing (*p* = 0.50, Tukey's test). Significant differences were noted in all other combinations (*p* < 0.05).

## Discussion/Conclusion

In this study, the ease and efficiency of four different cleaning techniques for washing the junctions of gastric nutrition tubes were evaluated. No significant differences were observed in bacterial colony counts after 28 days in any of the cleaning groups. The bacterial counts did not decrease, and it was concluded that bacterial growth could not be inhibited by washing once daily for 28 days. We previously reported the temporal changes in bacterial contamination of a route using a new and old standard [5]. The bacterial count in the route exceeded 10^4^ CFU/mL when using both the old and new standards [6, 7]; this was likely to be responsible for diarrhea on day 8, and no changes in the bacterial colony counts were observed at the endpoint (day 14). These results suggested that shifting to new standards may not increase the risk of bacterial adverse events. In our previous study, routes other than the nasogastric tube (e.g., a feeding bottle) were washed using several methods, but the number of bacteria in the routes did not decrease regardless of the washing method. In the present study, the bacterial colony counts exceeded 10^4^ CFU/mL on day 7, suggesting that the bacterial growth rate does not differ significantly between the entire route and the tube junction only.

The number of bacteria on day 7 was higher in experiment 2 than in experiment 1. The environment used for injection and cleaning differed between the experiments, which possibly affected the results. Humidity may also have affected bacterial growth [8]. However, a previous study, conducted at two different temperatures, found that the bacterial counts are not affected by temperature [9]. Reportedly, water temperature does not substantially affect bacterial counts [10]. Although the researchers who performed the experiments were the same, if the proficiency level of the experimental technique is not taken into consideration, it cannot be denied that the temperature and humidity of experiment 2 were higher than those of experiment 1 during nutrient administration, which contributed to the stable growth of bacteria.

Although we have not identified the bacterial species this time, several bacterial species, such as *Klebsiella aerogenes, Staphylococcus epidermidis*, and *Enterobacter cloacae*, were detected in our previous studies (personal communication; JSPEN. 2020 Nov; 2(5):316–26). Furthermore, no significant differences were observed in the bacterial counts between washing and control groups. As each group was washed only with water, it is possible that the nutrients could not be completely washed away [11]. High bacterial counts have been found in feeding tubes as a result of retrograde growth of bacteria on the outside of the tubes [12]. Overall, none of the washing processes helped decrease bacterial growth at the tube junctions. Moreover, no remarkable differences may have been observed among the washing groups, owing to the redeposition of bacteria [13]. However, a difference was observed in the appearance of the feeding tube junctions. In the control group, it was difficult to tighten the lids because of the nutritional residue (online suppl. Fig. 1d). Cleaning was also necessary to avoid leaving a bad impression on the patients.

The efficiency of cleaning was examined using the number of washes required. In experiment 2, water visually cleaned the tube junction with significantly fewer washes than required during experiment 1. Water washing appears to be the most effective method, given that none of the methods had any effect on the bacterial colony counts, and there was a high probability of effectively cleaning the tube junction with minimal washes using this method. It is important to minimize the number of washes and reduce the time and effort required for cleaning.

The number of washes with water required for adequate cleaning differed significantly between experiments 1 and 2. It is assumed that the number of vibrations per second and power of the vibration during water washing differed between experiments 1 and 2. In water washing, the connection is submerged in water and vibrated. From this, it is inferred that the cleaning mechanism is the flow of water and friction among the water, air bubbles, and the device. This is thought to be similar to the mechanism by which waves rub against a solid surface for cleaning with running water or ultrasonic waves in water [14]. Ultrasonic cleaning for 15 min removed 88.4% of *Candida* species; the CFU/mL count significantly declined during the first 10 min [15]. However, the cleaning efficiency of ultrasonic waves is not dependent on the frequency used [16]. Although the 1 s duration of vibration was not strictly specified, the degree of cleaning has been reported to reach an apparent equilibrium within a few minutes when fibers are cleaned with ultrasonic waves [16]. It is possible that even a few seconds were sufficient for the effect. Although the cleaning effect has been examined every few minutes in a previous study [17], no study has examined this effect within a few seconds. Water cleaning was easier to conduct than cleaning with cotton swabs. The choice of cleaning method should be based on what suits the actual situation. Although fibers and solid surface (grape surface in a previous study [16]) are more easily cleaned by increasing the number of washes with detergent [16, 17], we observed that the number of washes did not affect the bacterial counts. Sterile purified water was used for washing, but the number of bacteria did not decrease. In the future, measuring the use of hypochlorous acid solution and observing the cleaning effect will be considered.

One of the limitations of this study was that the control group was not included in experiment 1. As the bacterial count was measured experimentally as an indicator, it is not clear what effect contamination of the junction would have in clinical practice. We will investigate the adverse bacterial events in patients who have actually used the new standards in the future. We have not yet found any international reports pointing out the problems that have arisen after the change to the ISO standard.

Overall, this study found that the number of residual bacteria in the ISO-standardized tube junction was the same as without cleaning, regardless of the cleaning method applied. Furthermore, this study revealed potential problems associated with bacterial contamination.

## Statement of Ethics

As this study did not use any animals or cell cultures, the need for ethical approval was waived by the SHOWA University Clinical Research Review Board and the Graduate School of Pharmacy Ethics Committee on Research for Humans.

## Conflict of Interest Statement

The authors declare no potential conflicts of interest regarding this study.

## Funding Sources

We used research funds distributed to the Division of Social Pharmacy by SHOWA University. We did not receive any funds from other organizations or individuals.

## Author Contributions

Hiroaki Koya conducted the experiments and wrote the original draft of the manuscript. Keiko Ishino advised on the bacteriological methods that were used in this study and revised the draft from a bacteriological point of view. Makoto Kishimoto confirmed that the cleaning methods used for the experiments were not dissociated from those used in clinical practice and checked the draft from the viewpoint of clinical practice. Naomi Kurata helped design the experiments and proofread the entire draft.

## Data Availability Statement

The data supporting this study are available from the corresponding author upon reasonable request.

## Supplementary Material

Supplementary data

Supplementary data

## Figures and Tables

**Table 1:** The list of instruments and reagents used in the experiments

Use	Name	Manufacturer
Feeding syringe	NEOFEED Plastic Syringe (30 mL)	TOP Corporation
Feeding catheter	NEOFEED Feeding Catheter (14-Fr. 120 cm)	TOP Corporation
Enteral formula	RACOL^®^-NF Semi Solid for enteral use	Otsuka Pharmaceutical Factory, Inc.
Sterile petri dish	Acept Petri Dish	AS ONE Corporation
Sterile gauze	Sterile Gauze Sterile purepeido P	YAMATOKOJO CO., LTD.
Cleaning tools	EnClean^®^	LumaClean, LLC
	LISTERINE^®^ total care plus	Johnson & Johnson Services, Inc.
	Plush Cup 2 oz.	SUNNAP
	Toothbrush	
	Pediatric cotton buds (ultrafine)	matsukiyo
Medium	Brain heart infusion	Difco Laboratories
Sample storage container	Cryogenic Tubes 1.0 mL	Thermo Scientific™ Nunc™
