# Supplementary material for: Comparison of Methods for Cleaning Enteral Feeding Tube Junctions of the New International Standard (ISO 80369-3)
Source: Ann Nutr Metab. 2022 Jun 21;78(4):207–12. doi: 10.1159/000525367 (PMC9677827; doi:10.1159/000525367)
Supplement: Supplementary file 1 — Supplementary data [file anm-0078-0207-s01.pptx]

## Slide 1
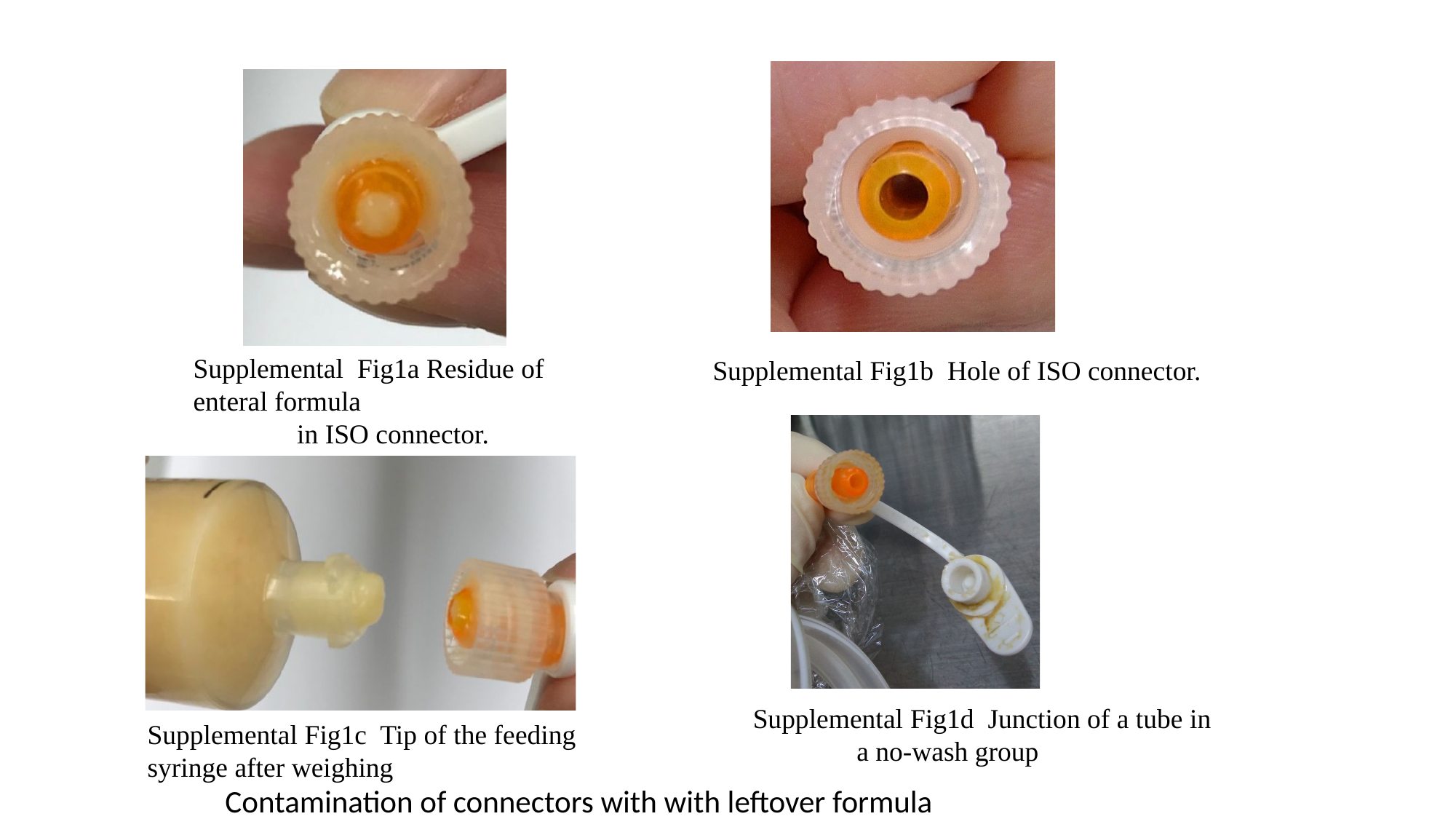

Supplemental Fig1a Residue of enteral formula
 in ISO connector.
Supplemental Fig1b Hole of ISO connector.
Supplemental Fig1d Junction of a tube in
 a no-wash group
Supplemental Fig1c Tip of the feeding syringe after weighing
Contamination of connectors with with leftover formula

## Slide 2
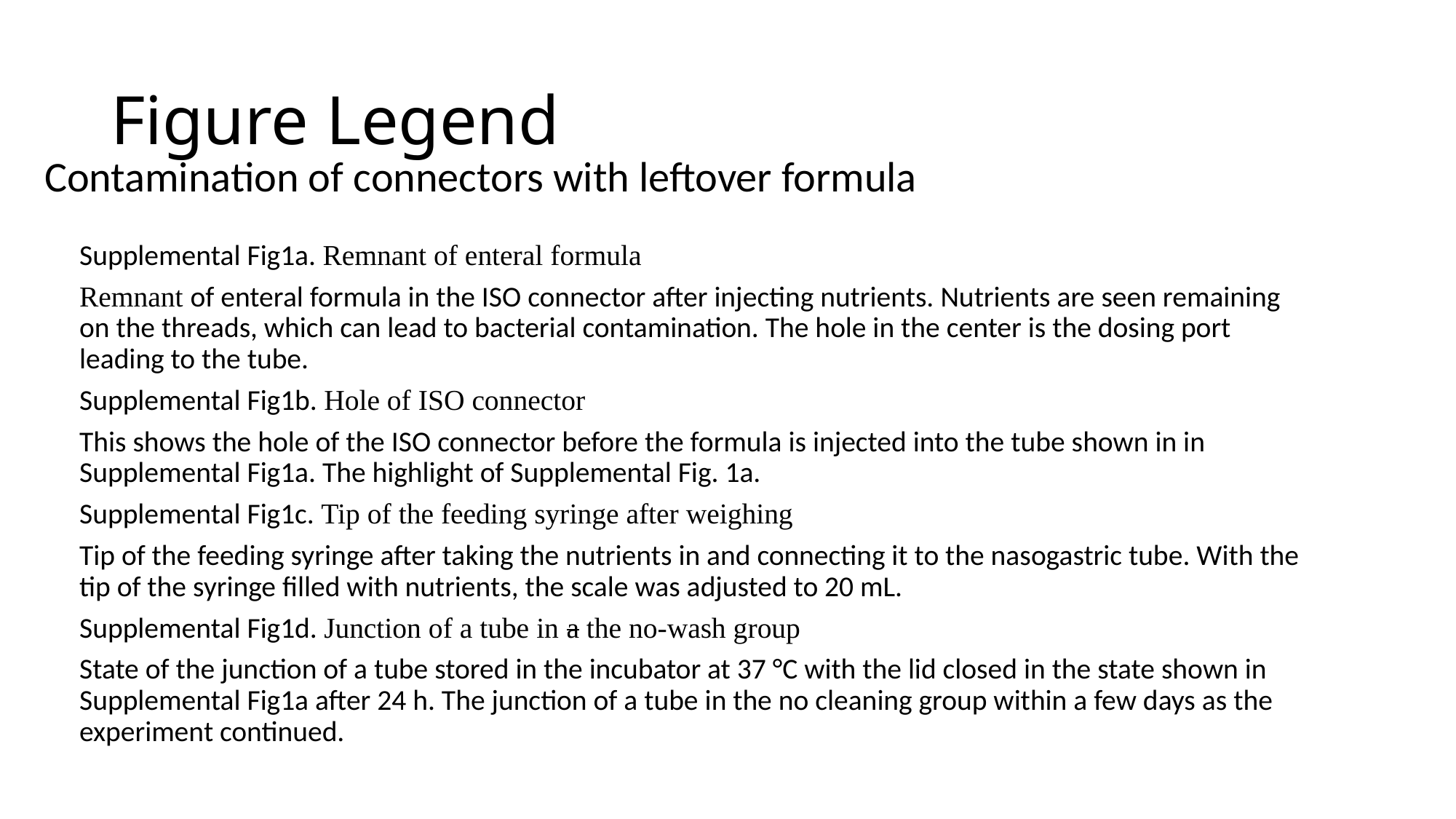

# Figure Legend
Contamination of connectors with leftover formula
Supplemental Fig1a. Remnant of enteral formula
Remnant of enteral formula in the ISO connector after injecting nutrients. Nutrients are seen remaining on the threads, which can lead to bacterial contamination. The hole in the center is the dosing port leading to the tube.
Supplemental Fig1b. Hole of ISO connector
This shows the hole of the ISO connector before the formula is injected into the tube shown in in Supplemental Fig1a. The highlight of Supplemental Fig. 1a.
Supplemental Fig1c. Tip of the feeding syringe after weighing
Tip of the feeding syringe after taking the nutrients in and connecting it to the nasogastric tube. With the tip of the syringe filled with nutrients, the scale was adjusted to 20 mL.
Supplemental Fig1d. Junction of a tube in a the no-wash group
State of the junction of a tube stored in the incubator at 37 °C with the lid closed in the state shown in Supplemental Fig1a after 24 h. The junction of a tube in the no cleaning group within a few days as the experiment continued.
