# Supplementary material for: Comparison of Methods for Cleaning Enteral Feeding Tube Junctions of the New International Standard (ISO 80369-3)
Source: Ann Nutr Metab. 2022 Jun 21;78(4):207–12. doi: 10.1159/000525367 (PMC9677827; doi:10.1159/000525367)
Supplement: Supplementary file 2 — Supplementary data [file anm-0078-0207-s02.pptx]

## Slide 1
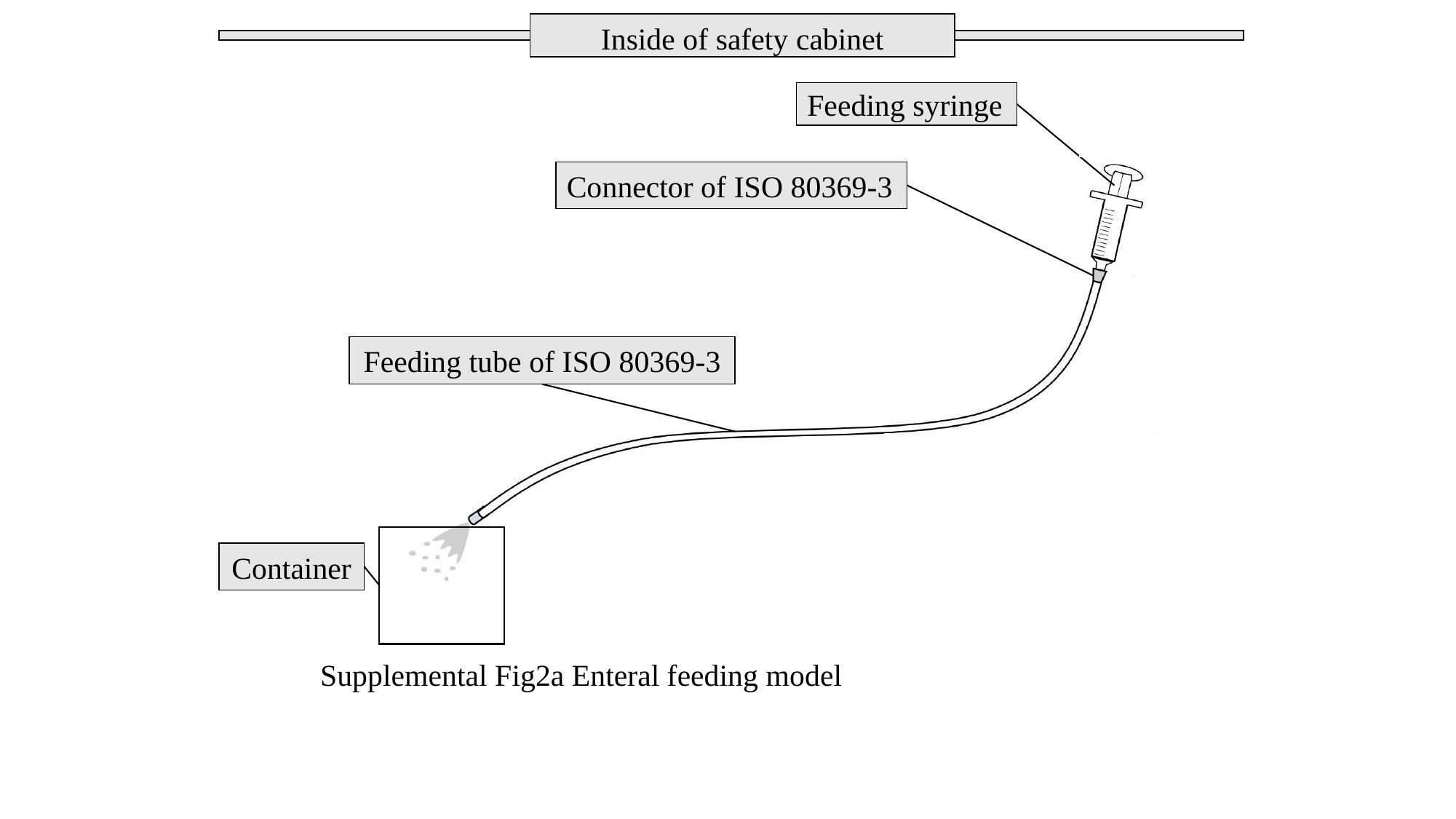

Inside of safety cabinet
Feeding syringe
Connector of ISO 80369-3
Feeding tube of ISO 80369-3
Container
Supplemental Fig2a Enteral feeding model

## Slide 2
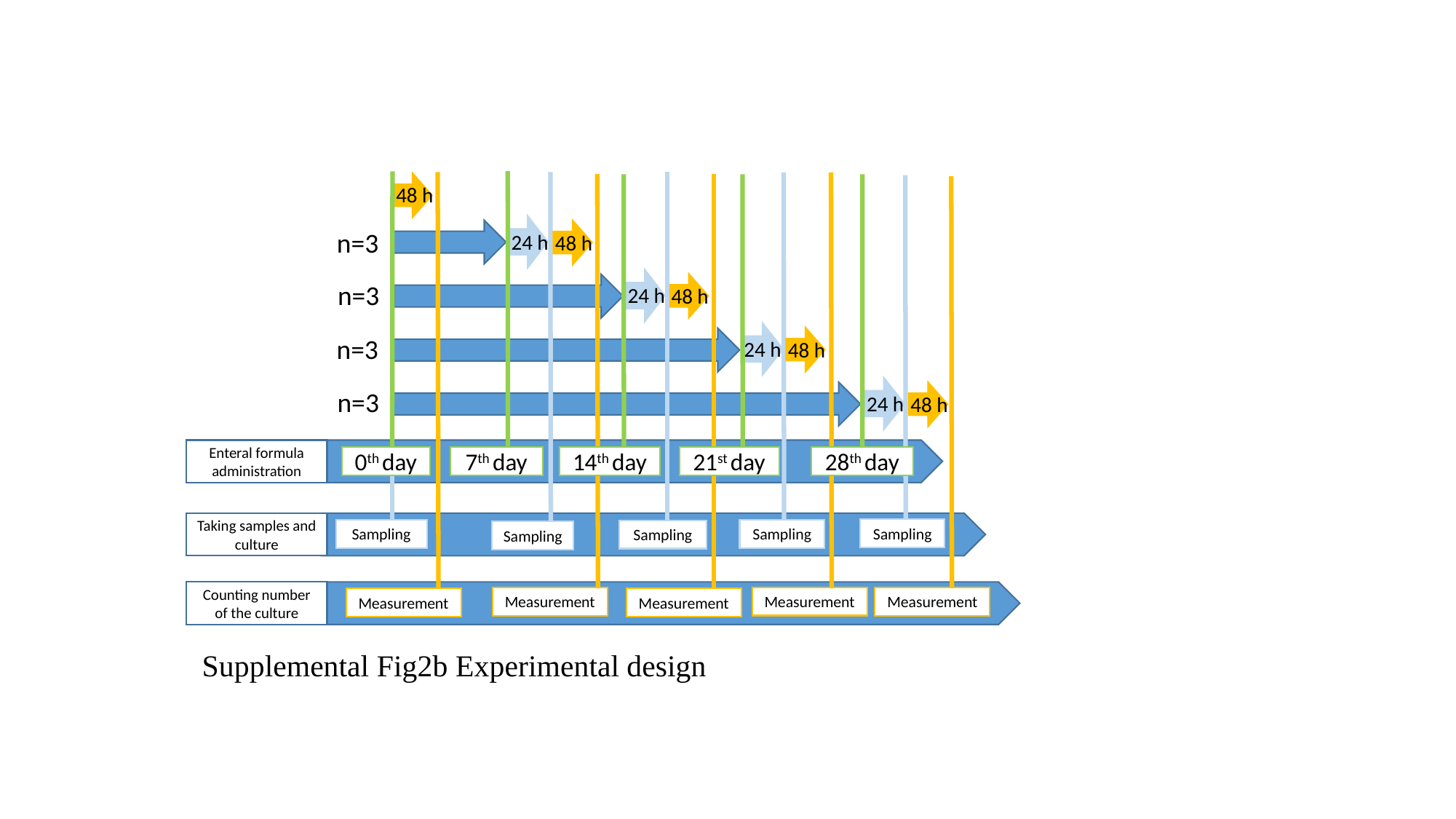

48 h
n=3
24 h
48 h
n=3
24 h
48 h
n=3
24 h
48 h
n=3
24 h
48 h
Enteral formula administration
14th day
28th day
21st day
7th day
0th day
Taking samples and culture
Sampling
Sampling
Sampling
Sampling
Sampling
Counting number of the culture
Measurement
Measurement
Measurement
Measurement
Measurement
Supplemental Fig2b Experimental design

## Slide 3
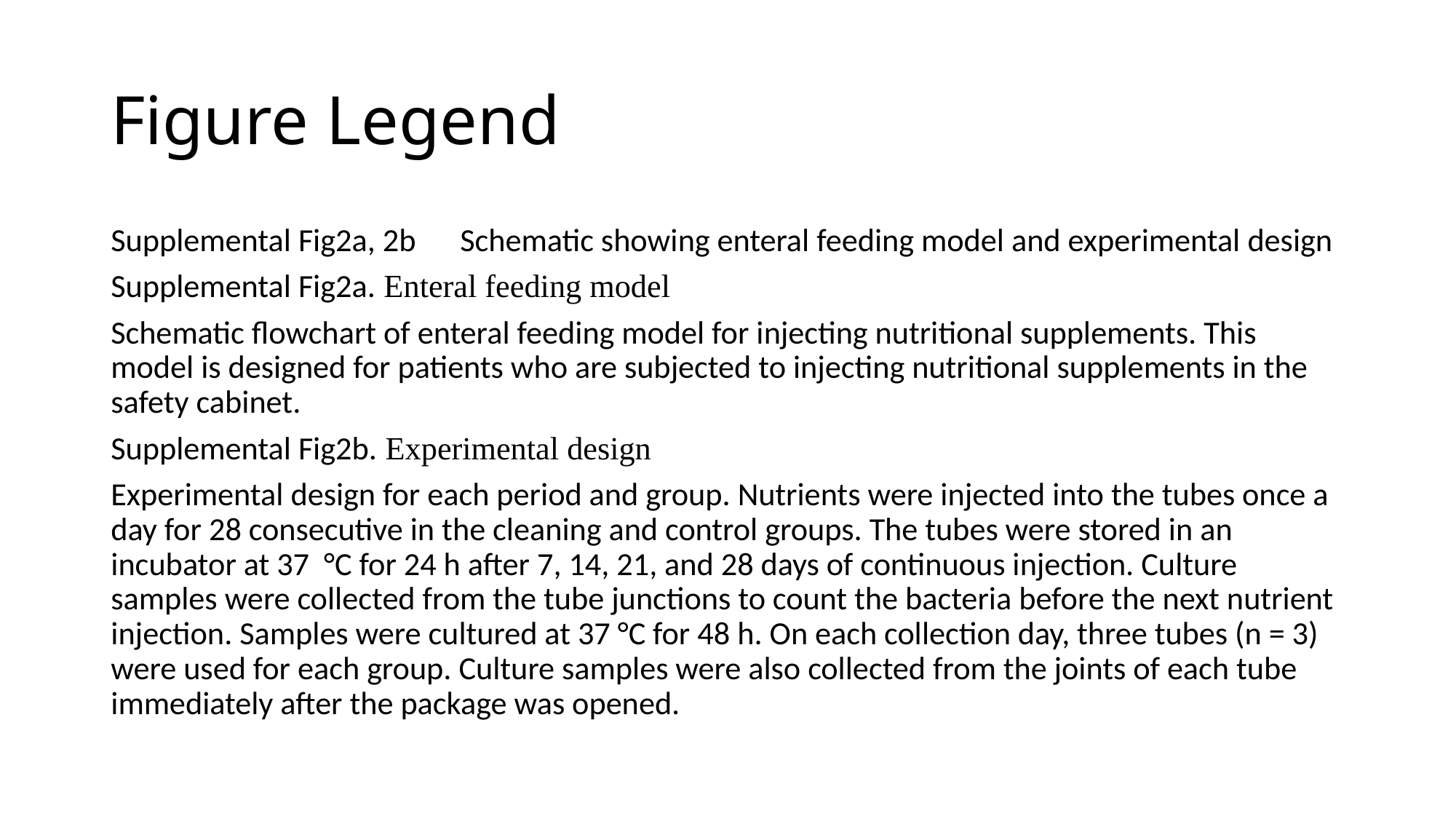

# Figure Legend
Supplemental Fig2a, 2b　Schematic showing enteral feeding model and experimental design
Supplemental Fig2a. Enteral feeding model
Schematic flowchart of enteral feeding model for injecting nutritional supplements. This model is designed for patients who are subjected to injecting nutritional supplements in the safety cabinet.
Supplemental Fig2b. Experimental design
Experimental design for each period and group. Nutrients were injected into the tubes once a day for 28 consecutive in the cleaning and control groups. The tubes were stored in an incubator at 37 °C for 24 h after 7, 14, 21, and 28 days of continuous injection. Culture samples were collected from the tube junctions to count the bacteria before the next nutrient injection. Samples were cultured at 37 °C for 48 h. On each collection day, three tubes (n = 3) were used for each group. Culture samples were also collected from the joints of each tube immediately after the package was opened.
